# Case report: Primary cardiac angiosarcoma with multiple metastases

**DOI:** 10.3389/fcvm.2022.941967

**Published:** 2022-07-28

**Authors:** Xuan Li, Lan Lan, Huijuan Hu

**Affiliations:** Department of Radiology, Zhongnan Hospital of Wuhan University, Wuhan, China

**Keywords:** cardiac angiosarcoma, metastasis, PET/CT, MRI, treatment

## Abstract

This reports outlines a rare case of primary right atrial angiosarcoma with multiple metastases. Multimodality imaging and histopathology confirmed the diagnosis of primary cardiac angiosarcoma and multiple metastases. We present the details of the presentation, multimodality imaging findings, and clinical management. The patient was followed up by cardiac MRI (CMRI) 2 months after therapy, the cardiac tumor and pulmonary metastases decreased markedly. Up to now, the patient has undergone four cycles of chemotherapy and immunotherapy.

## Introduction

Primary cardiac angiosarcoma (PCA) is extremely rare, and accounts for 33% of all primary malignant cardiac neoplasms (1). It originates from mesenchymal angioblasts and histologically it is composed of irregularly shaped vascular channels lined by anaplastic epithelial cells with large areas of intratumoral hemorrhage and necrosis (2). Clinical manifestations are not specific and depend on their infiltration into the myocardium and adjacent structures, as well as the extent of metastases (3). Patients usually visit a hospital because of chest pain, arrhythmia, peripheral edema, dyspnea, orthopnea, congestive heart failure, and pericardial tamponade (4). The most common location is the right atrium (RA). The prognosis of PCA is considerably poor, with a median survival of 14 months, reduced to 6 months in metastatic disease (5).

## Case presentation

A 28-year-old man was admitted to the orthopedics department on 13 January 2022 with low back pain for one month. On admission, the patient had neither previous cardiovascular antecedents nor family history. He also denied medication use. The patient's body temperature was normal, 36.6°C with a blood pressure of 102/68 mmHg, and a heart rate of 96 bpm. The patient body mass index was 20.2.

The biochemical investigation showed no alteration in the following serum levels: total bilirubin, alkaline phosphatase, urea, creatinine, potassium, glucose, and sodium. Hemoglobin level was 144 g/l, hematocrit level was 43.2%, and serum leukocyte and platelet levels were 9.2 × 10^9^/L and 165 × 10^9^/L, respectively.

Physical examination revealed pain in the sternocostal and lumbosacral region, other body systems showed no abnormalities. Electrocardiography was normal.

### Radiological investigations

**Figure 1:**
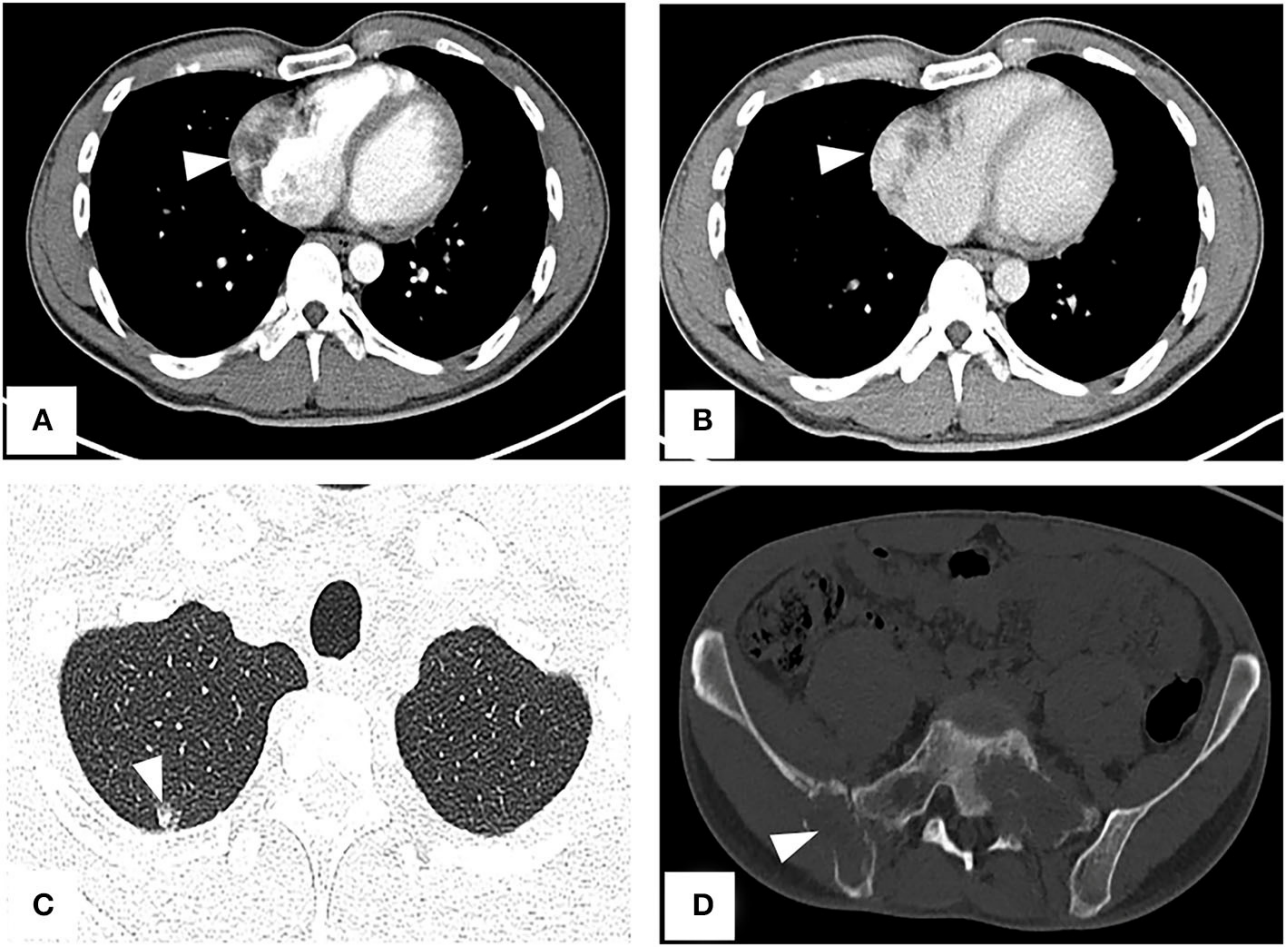
CT from chest to pelvis with enhancement shows right atrial tumor, small ground-glass pulmonary nodule, and bone destructions. **(A)** Arterial phase imaging reveals a filling defect in the right atrium (arrowhead). **(B)** Venous phase imaging shows the tumor inhomogeneously enhancing (arrowhead). **(C)** A lung window image shows a metastatic nodule in the upper right lung. **(D)** Bone destructions in the right ilium and sacrum.

**Figure 2:**
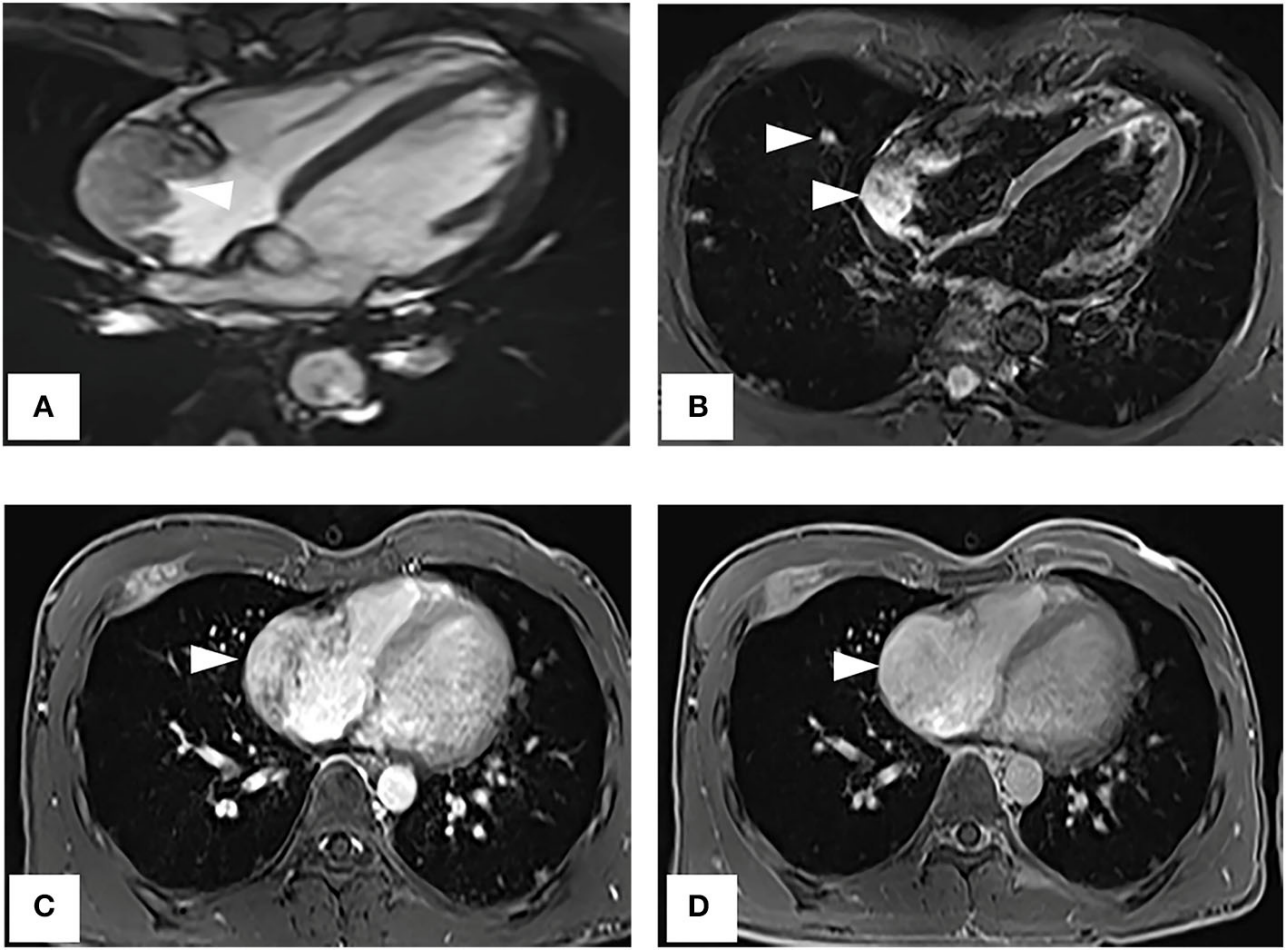
Cardiac magnetic resonance imaging. **(A)** On SSFP cine imaging planned in the four chambers, the tumor shows isointense (arrowhead). **(B)** On T2-short tau inversion recovery sequence images, the tumor displays high-signal intensity (arrowhead). **(C,D)** On enhanced MR imaging, the tumor shows arterial heterogeneous enhancement and progressive but incomplete enhancement in the delayed phase (dynamic acquisition).

**Figure 3:**
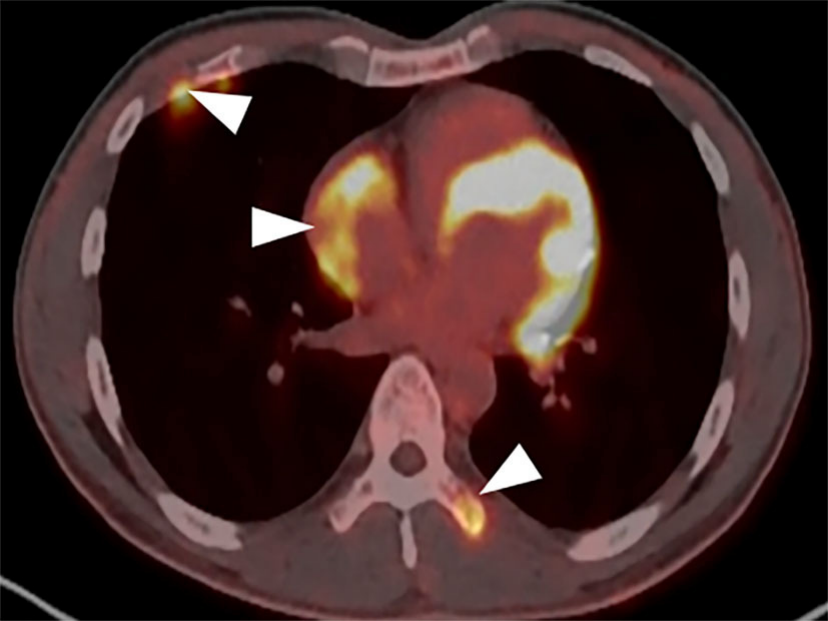
PET-CT demonstrates the tumor intensely increased FDG uptake with bony metastases (arrowhead).

A CT examination from the chest to the pelvis was performed, which revealed a right atrial mass, multiple small ground-glass pulmonary nodules, and bone destructions (Figures 1A–D). Transthoracic echocardiography (TTE) showed an ill-defined hypoechoic mass of approximately 3.8 cm × 2.3 cm, attached to the lateral wall of the right atrium. Cardiac MRI (CMRI) exhibited a right atrial tumor and multiple pulmonary lesions. On steady-state free precession (SSFP) cine imaging planned in the four chambers, the tumor showed isointense (Figure 2A). On T2-short tau inversion recovery (STIR) sequence images, the tumor displayed high-signal intensity (Figure 2B). On enhanced MRI, the tumor showed arterial heterogeneous enhancement and progressive but incomplete enhancement in the delayed phase (dynamic acquisition) (Figures 2C,D). The patient was referred for 18F-fluorodeoxyglucose (FDG) positron emission tomography-CT (PET-CT) for further characterization of the cardiac mass and systemic evaluation. PET-CT images demonstrated that the right atrial tumor had intensely increased FDG uptake (standardized uptake value, SUVmax, 8.4) with signs of pulmonary and bony metastases (Figure 3). These preoperative images characterized the mass as highly suspicious for a malignant cardiac tumor with multiple metastases.

### Management

**Figure 4:**
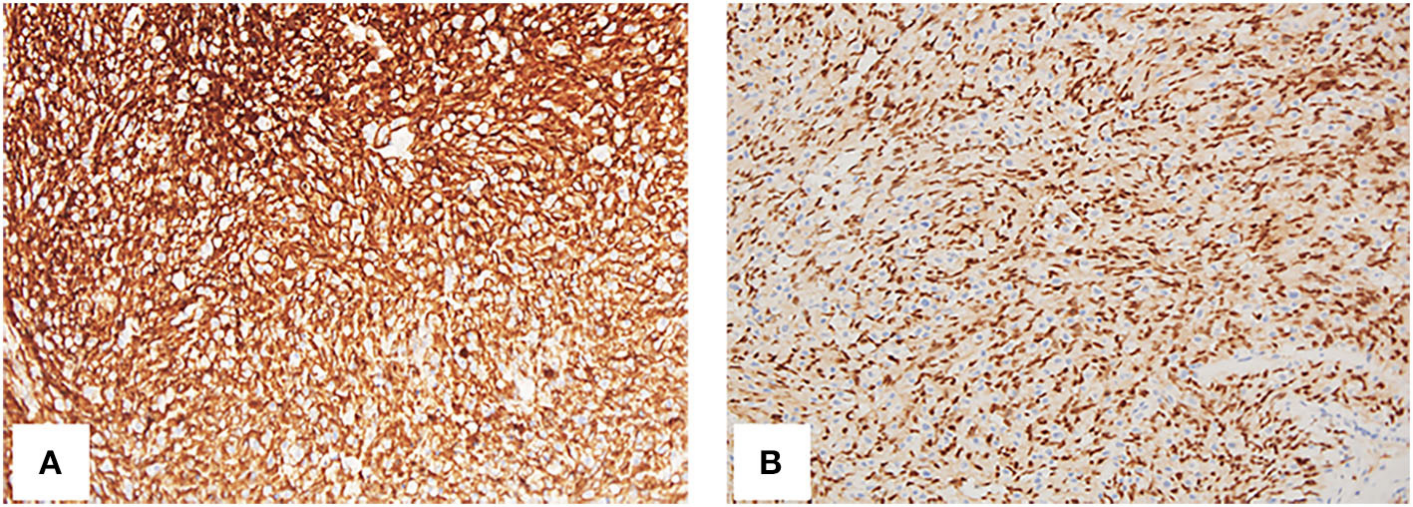
Histology of resection from the left lower lung nodule. The spindle cells are CD34 (**A**, 200 ×) and ERG (ETS-related gene) (**B**, 200 ×) positive.

To get a definitive diagnosis, the patient underwent the sacral biopsy, but the result was a benign vascularized lesion, which was in contradiction with the presentations on images, so the left lower lung nodule resection was performed. The histopathology examination and immunohistochemical staining supported the diagnosis of angiosarcoma positive for CD31, CD34, ETS-related gene (ERG), and Ki67 (LI: 60%) (Figures 4A,B). Combined with multimodality images, the final diagnosis was primary right atrial angiosarcoma with multiple metastases. The case was discussed in a multidisciplinary heart team meeting. Because of the presence of widespread metastases, curative treatment was considered to be impossible. The clinician suggested palliative chemotherapy and immunotherapy, and the patient agreed with the suggestion. Thus, the patient underwent chemotherapy and immunotherapy with epirubicin (10 mg, QD), ifosfamide (.5 g, QD), and pembrolizumab (100 mg, QD). Simultaneously the patient underwent palliative radiotherapy (20 Gy/5f) at the sites of the 10th thoracic vertebra. Up to now, the patient has undergone four cycles of treatment.

### Follow-up period

**Figure 5:**
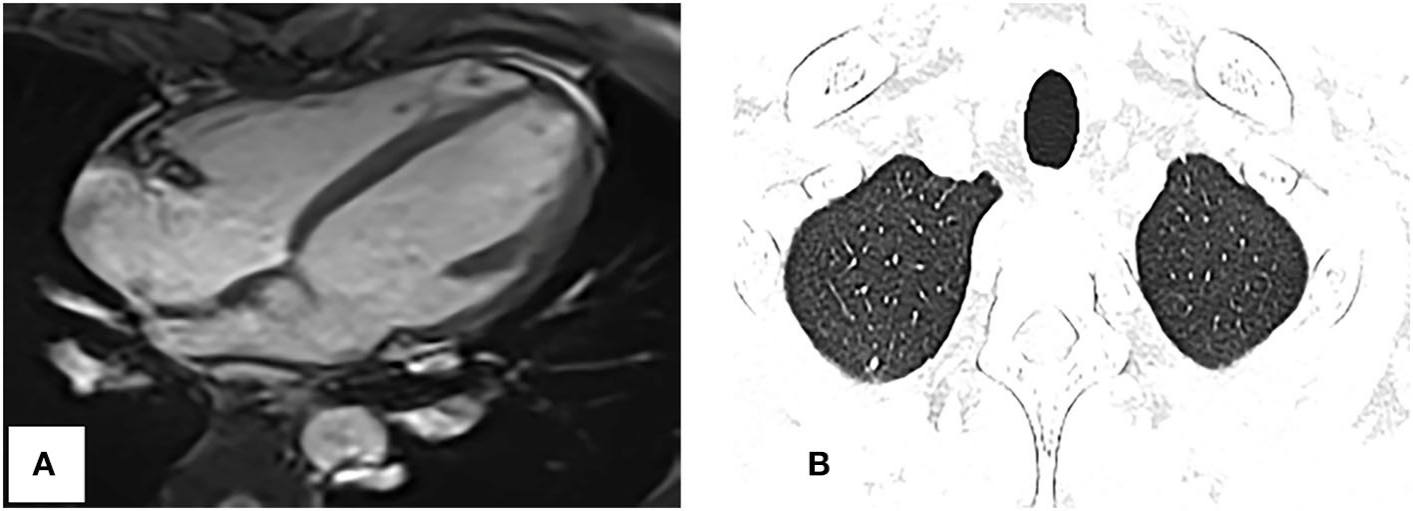
Repeat cardiac magnetic resonance imaging and thoracic CT. **(A)** SSFP cine imaging planned in the four chambers shows the tumor markedly reduced. **(B)** Thoracic CT shows the pulmonary nodules obviously decreased too, compared with Figure 1C.

Repeat CMRI and chest CT showed significant regression of the tumor and pulmonary metastases (Figures 5A,B). Now, the patient continues the chemotherapy and immunotherapy.

## Discussion

PCA is one of the most common primary cardiac malignancies (3), originated preferentially in the right atrium (approximately 90%), while <5% occur in the left atrium or ventricles (6). PCA commonly affects male patients those 30–40 years of age as in this case (7). The clinical symptoms are non-specific, depending on location and size, such as chest pain, chest tightness, shortness of breath, arrhythmia, pericar-dial effusion, or right heart failure (8, 9). The overall prognosis is extremely poor due to the aggressive and frequent presence of metastases at the time of diagnosis (3, 8–10).

Echocardiography has become an initial imaging technique because of its wide availability and high sensitivity, non-invasiveness, and cost-effectiveness (8, 11, 12), but it has some limitations, such as operator dependence, and the views may be limited in some patients (13).

There is more efficiency in using CT for tissue characterization and systemic metastases, especially in the lungs (3). PCA often presents as homogeneous or inhomogeneous density on unenhanced CT scans and heterogeneous centripetal enhancement on enhanced images (14).

Cardiac MRI is considered to be the most sophisticated imaging method to display the cardiac tumor size, location, and signal characteristics (15). T1-weighted images show tumor as predominantly isointense to the myocardium and high signal on T2-weighted images. Late gadolinium-enhanced (LGE) is avid and may predominate along prominent vascular channels to give a characteristic “sunray” pattern (2). In our case, CMRI clearly showed the tumor-infiltrating the free wall of the RA with obvious heterogeneous enhancement indicative of malignancy.

Positron emission tomography/CT has advantages in non-invasively characterizing cardiac tumors and disease staging, scarce case reports have described the use of FDG PET-CT in differentiating the benign and malignant mass, metastatic workup, preoperative staging, and assessment of response to treatment in primary cardiac angiosarcomas (16–19). Rahbar et al. (20) in their study of various cardiac tumors proposed that malignancy was determined with a sensitivity of 100% and specificity of 86% (accuracy, 96%) after a cutoff with high sensitivity (SUVmax of 3.5) was chosen to avoid false-negatives. In our case, the SUV max of the tumor is 8.4, which is highly suspicious of malignancy.

Although multimodality images can help diagnosis, definitive diagnosis and classification depend on the pathological examination, which is the gold standard based on immunohistochemistry as evidence for chemotherapy and radiotherapy. Immunohistochemically, angiosarcomas are usually positive for vimentin, CD31, CD34, and factor VIII-related antigens (21). ERG was the most sensitive vascular marker, with diffusely reactive in all cases of cardiac angiosarcoma and its superior sensitivity to CD31 and CD34 (22). In our patient, CD31, CD34, and ERG were positive, which supported the diagnosis of angiosarcoma.

The most common organ for metastasis of primary angiosarcoma is the lungs and bone, occasionally in the liver and spleen, and extremely rarely in the brain (14, 23). Pulmonary metastases manifested different sized multiple hemorrhagic nodules (24), which was a characteristic CT appearance, consisting of a central area of soft-tissue attenuation surrounded by a halo of ground-glass attenuation. This halo sign was previously established as a common finding correlated with pulmonary angiosarcoma, and it also helps in detecting metastatic pulmonary lesions (23). In this report, this case had typical halo signs on thoracic CT.

Due to the rarity of these tumors, the optimal management strategy is still argued. Current treatment options include surgery, radiotherapy, and chemotherapy. Surgical resection remains the gold standard for the treatment of primary malignant cardiac tumors. The limits of surgical resection have been related to the degree of extensive involvement of nearby structures and other cardiac chambers (25). In addition, targeted medicines and immunotherapy have been studied as promising treatments for angiosarcoma (26). Although the benefits of adjuvant therapy and other strategies are still unknown, there is a tendency to use more aggressive treatment for younger patients (25). Because of widespread metastases, our patient received adjuvant immunotherapy and chemotherapy, which has a good effect. Close follow-up is mandatory because of the poor prognosis.

There were limitations in this study. First, the patient did not conduct surgical resection, and no pathology of the tumor was obtained. We speculated the diagnosis according to histopathologic findings of lower left lung and multimodality images. Second, at present adjuvant immunotherapy and chemotherapy is very effective, but we should follow up closely to observe the future effect.

## Conclusion

Evaluation and diagnosis of PCA can be complex and should include multimodality imaging, multidisciplinary discussion is important. The case demonstrates the typical presentation of primary cardiac angiosarcoma and its associated features on multimodality imaging. More experience is needed to better understand the imaging characteristics of angiosarcoma.

## Data availability statement

The original contributions presented in the study are included in the article/supplementary material, further inquiries can be directed to the corresponding author.

## Ethics statement

The studies involving human participants were reviewed and approved by Medical Ethics Committee of Zhongnan Hospital of Wuhan University. The informed consent was waived.

## Author contributions

XL contributed to MRI scanning of the patient and collecting clinical data. HH contributed to editing, revising, and approving the manuscript. LL contributed to interpreting the data. XL, HH, and LL contributed to the writing and revision of the manuscript. All authors have read and approved the final manuscript.

## Conflict of interest

The authors declare that the research was conducted in the absence of any commercial or financial relationships that could be construed as a potential conflict of interest.

## Publisher's note

All claims expressed in this article are solely those of the authors and do not necessarily represent those of their affiliated organizations, or those of the publisher, the editors and the reviewers. Any product that may be evaluated in this article, or claim that may be made by its manufacturer, is not guaranteed or endorsed by the publisher.
